# Investigating neuropsychological and reward-related deficits in a chronic corticosterone-induced model of depression

**DOI:** 10.1016/j.psyneuen.2022.105953

**Published:** 2023-01

**Authors:** Claire A. Hales, Sarah A. Stuart, Jennifer Griffiths, Julia Bartlett, Roberto Arban, Bastian Hengerer, Emma SJ Robinson

**Affiliations:** a University of Bristol, School of Physiology, Pharmacology & Neuroscience, Biomedical Sciences, University Walk, Bristol BS8 1TD, UK; b CNS Diseases Research, Boehringer Ingelheim Pharma Gmbh & Co. KG, Biberach an der Riss, Germany; c Department of Psychology, Djavad Mowafaghian Centre for Brain Health, University of British Columbia, 2215 Wesbrook Mall, Vancouver, BC V6T 1Z3, Canada

**Keywords:** Affective biases, Memory, Reward, Decision-making, Corticosterone, Stress

## Abstract

Chronic stress is a known risk factor for the development of major depression (MDD) and is commonly used to induce a depression-like phenotype in rodents. Similar phenotypic effects are also observed in rodents when treated chronically with the stress hormone corticosterone. In this study, we investigated the neuropsychological consequences of chronic corticosterone treatment in male rats using two translational rodent assays of affective bias, the judgement bias task (JBT) and affective bias test (ABT). We also used the reward learning assay (RLA) and sucrose preference test (SPT) to quantify reward-related behaviours. Negative biases in decision-making were observed in the chronic corticosterone-treated group but only when the treatment was given shortly before each behavioural session. The same dose of corticosterone, when given daily after completion of the behavioural session had no effects. Chronic corticosterone treatment did not potentiate negative affective biases in the ABT induced by either an acute pharmacological or stress manipulation but both reward learning and reward sensitivity were blunted. Analysis of the brain tissue from animals receiving chronic corticosterone found reduced hippocampal neurogenesis consistent with previous studies suggesting corticosterone-induced neurotrophic deficits. Taken together, these data suggest chronic corticosterone treatment induces neuropsychological effects related to changes in reward learning, memory and negative biases in decision making, but these decision-making biases depend on whether rewarding outcomes were experienced during the acute effects of the drug. These findings suggest an important interaction between psychological and biological factors resulting in negative biases in decision-making in this model.

## Highlights

• Chronic corticosterone (CORT) treatment induced negative decision-making biases.
• Negative decision-making biases were dependent on treatment timing.
• Chronic CORT treatment caused impairments in reward learning and reward sensitivity.
• CORT treatment did not potentiate pharmacological or stress induced negative bias.
• Chronic CORT treatment reduced neurogenesis in the hippocampus.

## 1 Introduction

Our understanding of the aetiology of MDD is limited, however, epidemiological studies have identified significant negative psychosocial events, early life adversity, and/or a family history of MDD as risk factors for the disorder (Heim and Nemeroff, 2001, Sullivan et al., 2000). Chronic stress in particular is believed to be one of the most important clinical risk factors for MDD (Bruce, 2002, Bruce and Kim, 1992, Krishnan, 2002, Lorant et al., 2003, Yang et al., 2015, Otte et al., 2016). Chronic stress and chronic treatment with the stress hormone, corticosterone (CORT), have also both been used to generate depression-like phenotypes in rodents and are commonly used as a disease model for studying MDD (Gregus et al., 2005, Johnson et al., 2006, Willner, 2005, Zhao et al., 2008).

In recent years there has been renewed interest in a cognitive neuropsychological hypothesis of depression which proposes that negative biases in the processing of emotional (‘affective’) information play a key role in the development, maintenance and treatment of MDD (Elliott et al., 2011, Harmer et al., 2009, Roiser et al., 2012). Patients with MDD exhibit negative affective biases in multiple cognitive domains, including attention, learning and memory, emotional processing and interpretation of ambiguous affective information (Clark et al., 2009, Gotlib and Joormann, 2010, Mathews and MacLeod, 2005). Although not specifically associated with affective biases, impairments in reward learning, which dissociate the experience of pleasure and motivation for reward, are also thought to be a neuropsychological feature of MDD (Der-Avakian et al., 2016, Slaney et al., 2018). These affective biases and reward learning deficits can be measured in laboratory animals using ‘reverse translated’ tasks that measure similar neuropsychological processes as in humans (for reviews see Hales et al., 2014; Robinson and Roiser, 2016, Slaney et al., 2018). Specifically, the affective bias test (ABT) has been developed to study biases in reward learning and memory (Stuart et al., 2013), and the judgement bias task (JBT) has been developed to measure decision-making biases in the interpretation of ambiguous cues (Harding et al., 2004, Roelofs et al., 2016). The reward learning assay (RLA) is a modification of the ABT where different cue-reward values are learnt generating a reward-induced bias during a subsequent choice test (Robinson, 2018, Stuart et al., 2017, Stuart et al., 2019).

The majority of affective bias studies in rodents have involved normal animals and acute affective state manipulations, however we have previously published data suggesting that early life stress in rats increases vulnerability to negative affective biases and impairs their ability to appropriately learn reward value in the RLA (Stuart et al., 2019). In this early life adversity model we also observed a dissociation between reward learning impairments in the RLA and reward sensitivity in the sucrose preference test (SPT) or motivation in the progressive ratio task. Given the extensive preclinical use of chronic stress and chronic CORT models in rodents, in this study our aim was to investigate the neuropsychological changes which develop in this model. By integrating this established disease model with our novel translational tasks, our studies were designed to understand the neuropsychological deficits associated with a biologically induced chronic stress and depression-like state. We first investigated the effects of chronic CORT treatment in animals which had been trained in the JBT. Animals were tested daily with the treatment given after the behavioural sessions however, we failed to see any effects. Given that affective biases have been linked to learning and memory, we re-ran the treatment protocol but administered CORT before the behavioural session. By changing the timing of the treatment, peak levels of the CORT were present during the behavioural task and hence could have a greater impact on the animal’s experience of the task, associated learning and memory and hence future decision-making biases. We also used the ABT to test whether animals which had received chronic CORT treatment would show similar enhanced vulnerability to negative affective biases in the ABT, as seen in the early life adversity model (Stuart et al., 2019). In these same animals we also tested reward-related behaviours using the RLA and SPT. In addition to the chronic CORT treatment, we also ran a second cohort of animals for the ABT, RLA and SPT where the intensity of the stress manipulation was increased by including isolation housing as well as the chronic CORT treatment.

## 2 Materials and methods

### 2.1 Ethics statement

All procedures were conducted in accordance with the requirements of the UK Animals (Scientific Procedures) Act 1986 and in accordance with local institutional guidelines under a Home Office project licence. Experiments were conducted and are reported in line with the ARRIVE guidelines. During experiments all efforts were made to minimise suffering, and at the end of experiments rats were killed by giving an overdose of sodium pentobarbital (200 mg/kg).

### 2.2 Subjects

The animals used were four cohorts of 16 male Lister Hooded rats (Harlan, UK). For JBT experiments, rats were approximately 3 months in age and weighed 260–305 g (experiment 1) / 270–305 g (experiment 2) at the start of training, and 390–500 g (experiment 1) / 375–450 g (experiment 2) at the start of chronic CORT treatment. For ABT experiments, rats weighed 470–525 g (experiment 3) / 420–510 g (experiment 4) at the start of chronic CORT treatment. Animals were housed in pairs under temperature (19–23 °C) and humidity (45–65%) controlled conditions and a 12:12 h light–dark cycle (experiments 1–2: lights off at 0800 h; experiments 3–4: lights off at 0700 h). All animals were provided with environmental enrichment including nesting material, cardboard tube and red Perspex shelter. They were maintained at no less than 90% of their free-feeding weight by restricting access to laboratory chow (LabDiet, PMI Nutrition International) to ∼18 g per rat per day. Water was provided ad libitum. All behavioural testing was conducted between 0900 h and 1800 h during the animals’ active phase.

### 2.3 Experimental design and drugs

**Fig. 1:**
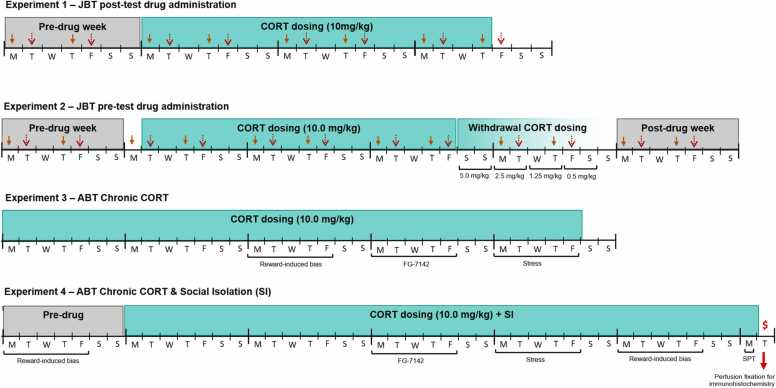
Schematic of the experimental design. Experiment 1 was made up of two parts: a pre-drug week, followed by three weeks of CORT dosing (10.0 mg/kg, s.c. or VEH; 0.0 mg/kg, s.c.). During the CORT dosing period, rats were dosed daily at least 3 h following the end of behavioural testing in the JBT, or at an equivalent time on days when testing did not occur (Wednesdays and weekends). Solid headed arrows denote a baseline behavioural testing session on the JBT; dashed open headed arrows denote an ambiguous probe testing session on the JBT. Experiment 2 was made up of four parts: (1) a pre-drug week, (2) three weeks of CORT dosing (10.0 mg/kg, s.c. or VEH; 0.0 mg/kg, s.c.), (3) CORT dosing on a reducing dose schedule (5.0, 5.0, 2.5, 2.5, 1.25, 1.25, 0.5, 0.5 over 8 days) to prevent CORT withdrawal, and (4) a post-drug week. Animals were dosed daily 30 min prior to behavioural testing in the JBT, or at an equivalent time on days when testing did not occur (Wednesdays and weekends). Solid headed arrows denote a baseline behavioural testing session on the JBT; dashed open headed arrows denote an ambiguous probe testing session on the JBT. In Experiment 3 animals received five weeks of CORT dosing (10 mg/kg s.c. or VEH; 0.0 mg/kg, s.c.) and were tested for reward-induced positive bias, FG7142-induced negative bias, and stress-induced negative bias. In Experiment 4, animals were tested for reward-induced positive bias in the week prior to commencing CORT dosing (10 mg/kg s.c. or VEH; 0.0 mg/kg, s.c.) with additional social isolation (SI) following behavioural testing sessions. Animals were then tested for FG7142-induced negative bias, stress-induced negative bias and reward-induced positive bias during CORT+SI treatment. A sucrose preference test (SPT) was carried out as indicated, and animals were killed ($) and the brains processed for DCX and Ki-67 immunohistochemistry to assess neurogenesis. M, T, W, T, F, S, S denotes days of the week (Monday, Tuesday, Wednesday, Thursday, Friday, Saturday, Sunday).

In all experiments, a between-subjects study design was used for the main treatment. In experiment 1, following completion of training on the JBT, a 7 day ‘pre-drug’ period was followed by 18 days of daily drug treatment with either corticosterone (CORT; 10.0 mg/kg) or vehicle (VEH, 0.0 mg/kg; Fig. 1). Experiment 1 was terminated after this chronic treatment period due to a lack of drug effect. In experiment 2, the same ‘pre-drug’ and CORT dosing period (18 days) were used and following an initial analysis of the data and observation of a main effect of treatment, an 8 day ‘withdrawal’ period was carried out and the animals then re-tested for a further 7 day post-treatment period. This was included to enable us to see if effects were sustained or reversed. The withdrawal period involved a reducing dose schedule (5.0, 2.5, 1.25, 0.5 mg/kg), each administered for two consecutive days in order to mitigate the physiological effects of withdrawal (Fig. 1). Behavioural testing using the JBT was carried out throughout the experimental periods. Rats were split into VEH or CORT groups based on performance on the JBT during the pre-drug week (matched for all analysed behavioural variables). Rats in experiment 1 were dosed daily at least 3 h following the end of behavioural testing, commencing the Monday of first drug week and ending on the Thursday of final drug week. Rats in experiment 2 were dosed daily 30 min prior to behavioural testing, with the first dose prior to the Tuesday probe test during the first drug week, and the final dose prior to the Friday probe test of the third drug week. In both experiments 1 and 2, dosing occurred at an equivalent time of day and may have been influenced by the natural circadian rhythm.

In experiment 3 (ABT, RLA and SPT) chronic CORT treatment consisted of a once-daily subcutaneous injection of 10 mg/kg CORT or VEH for control animals. Injections were administered at a random time between 09.00 and 17.00 h such that the treatment was unpredictable and less susceptible to natural changes in endogenous CORT levels. For experiment 4, the protocol was further adapted to include the addition of social isolation (CORT+SI) to add a psychosocial stress to the induction of a depression-like phenotype. In these studies CORT-treated animals were also housed individually in unenriched cages separated with paper partitions to prevent visual contact between animals. Vehicle-treated animals (VEH) remained in normal pair-housing conditions. In both experiments, chronic treatment started 14 days before behavioural testing using the ABT and continued for the experiment duration.

### 2.4 Experiments 1 & 2: judgement bias task

JBT testing was carried out using standard rat operant chambers (Med Associates, Sandown Scientific, UK). Operant chambers were configured as in Hales et al. (2017), (Hales et al., 2020). Rats were tested using a high vs. low reward version of the JBT as previously reported in Hales et al. (2017), (Hales et al., 2020). Rats were trained to associate a correct lever press response to two different auditory tones with either a high or low value reward. Responses made following a midpoint ambiguous tone were used to measure judgement bias.

### 2.5 Judgement bias training

Training was the same for both experiments: rats were trained to associate one tone (2 kHz at 83 dB rats, designated high reward) with the receipt of four 45 mg reward pellets (TestDiet, Sandown Scientific, UK) and the other tone (8 kHz at 66 dB, designated low reward) with a single 45 mg reward pellet if they pressed the associated lever (either left or right, counterbalanced across rats) during the 20 s tone. Table S1 contains a summary of training stages used. Rats completed training once they maintained stable responding for three consecutive days, but were excluded from analyses if they failed to maintain 60% accuracy on reference tones (high and low reward) during experiments. Once trained (29 total sessions for experiment 1, 24 sessions for experiment 2, see Table S1 for number of sessions required for each training stage), animals were used in experiments 1 and 2.

### 2.6 Judgement bias testing

Baseline sessions (100 trials: 50 high and 50 low reward tones; pseudorandomly) were conducted on Monday and Thursday. Probe test sessions (120 trials: 40 high reward, 40 low reward, and 40 ambiguous midpoint tones that were 5 kHz at 75 dB; pseudorandomly) were conducted on Tuesday and Friday. Both experiments tested the effect of chronic treatment with CORT on judgement bias, but differed in the timing of daily CORT treatment with respect to judgement bias testing: CORT treatment after behavioural testing in experiment 1; CORT treatment before (30 min pre-treatment) behavioural testing in experiment 2. In both cases, the midpoint tone was randomly reinforced so that a specific outcome could not be learnt for the midpoint tone, and to ensure continued responding throughout the experiments. For further task details see Supplementary Materials and Methods section of Hales et al. (2017).

## 3 Experiments 3 & 4: ABT and RLA testing and immunohistochemistry

### 3.1 ABT and RLA training and testing procedure

Before the chronic CORT procedure commenced, all rats were first habituated to a 40 cm^2^ Perspex test arena and trained to dig in two bowls filled with digging substrate (e.g., paper bedding, sawdust, cloth etc.) to obtain a quantity of food pellets (45 mg rodent tablet, TestDiet, Sandown Scientific, UK). Training was complete once each rat was able to find the pellets on 12 consecutive trials within 30 s for each trial. Once trained, each study followed a standard protocol of four pairing sessions followed by a preference test session on the fifth day. Each pairing session consisted of discrete trials in which the animal was placed into the testing arena and allowed to approach and explore two bowls, one rewarded substrate and the other unrewarded ‘blank’ substrate. Once the animal started digging in one bowl, the other was removed by the experimenter, the latency to dig recorded, and the trial recorded as correct (rewarded substrate) or incorrect (blank substrate). If the animal failed to approach the bowls and dig within 20 s, the trial was recorded as an omission. Animals were run until they completed six consecutive correct trials. All standard ABT studies used a within-subject design wherein each animal learnt to associate two different digging substrates (A or B) with a food pellet reward during pairing sessions. These pairing sessions were carried out on separate days following either a treatment manipulation or a vehicle/control manipulation. The pairing sessions were carried out on days 1–4 and, on day 5, the rats were presented with both reinforced substrates together for the first time and their choices over 30 trials recorded. For the preference test trials, a single pellet was placed in one of the bowls using a random reinforcement protocol such that there was a 1 in 3 probability for each substrate. Trials were run as described above, and the animals’ latency to dig and choice of substrate (A or B) was recorded. In all studies, the substrate, pairing session, and treatments (i.e. the manipulation used to induce a bias) were fully counterbalanced. Results from the preference test day were recorded as number of choices for the vehicle-paired substrate vs the number of choices for the treatment-paired substrate and were used to calculate a percentage choice bias value for further analysis.

In both experiment 3 and 4, animals ran one RLA and two ABTs. Firstly, to assess the animals’ ability to develop a bias based on absolute reward value, a reward-induced bias test was carried out using the RLA. In this study, one substrate was paired with two food pellets and the other substrate with a single food pellet. Preference testing then used a single pellet and random reinforcement. Previous tests of this nature show that normal rats develop a significant positive bias for the substrate associated with the higher value of reward (Stuart et al., 2013). Subsequently, the effect of chronic stress on the development of negative affective bias was tested using either the anxiogenic compound FG7142, or psychosocial stress. Both treatments have previously been shown to induce a significant negative affective bias in normal rats (Stuart et al., 2013). We used FG7142 as the animals were already being treated with chronic CORT, and so also using CORT treatment to generate an acute negative bias (as in our early life adversity animals) was potentially confounded. FG7142 is a benzodiazepine inverse agonist which generates an anxiogenic affective state following acute administration in rodent models and human studies (Evans and Lowry, 2007). To assess the effects of FG7142, one substrate was paired with 5 mg/kg FG7142, administered 30 min before the pairing session, and the other following vehicle treatment (vehicle). To assess the effects of acute stress, one substrate was paired following 10 min (experiment 3), or 30 min (experiment 4) of restraint stress, and the other substrate was paired without stress. In experiment 3 only, the stress pairing sessions were immediately followed with 5 h of isolation housing, and control sessions were followed by normal paired housing. This was not required in experiment 4 since the CORT+SI group was already housed in isolation for the duration of the study period.

### 3.2 Sucrose preference test

In experiment 4, a SPT was also conducted. The protocol for the SPT was adapted from previous studies (Banasr et al., 2007, Willner et al., 1987). Three days before the test, rats were given access to one bottle of water and one bottle of 1% sucrose solution in their home cage for 48 h. The sucrose solution was then replaced with water until the test day. On the test day the animals were deprived of water for 4 h and moved into clean individual test cages. Sucrose preference was then determined by a 2 h exposure to two identical bottles, one containing 1% sucrose and the other water. The position of the bottles was counterbalanced across animals and was switched at 30 min intervals during the test. Sucrose preference was defined as the ratio of the volume of sucrose vs. water consumed during the test.

### 3.3 Immunohistochemistry

Following the completion of the behavioural testing in experiment 4, and 24 h after the last CORT injection, the animals were anaesthetised with a lethal dose of sodium pentobarbital (0.5 ml Euthatal, 200 mg/ml, Genus Express, UK) and perfused via the left ventricle with 0.01 M phosphate buffered saline (PBS) followed by 4% paraformaldehyde. The brains were removed and post-fixed in paraformaldehyde for at least 24 h. Prior to being cut, the brains were transferred to 30% sucrose in 0.1 M PBS for 48 h. Coronal sections were cut at 40 µm on a freezing microtome and stored in cryoprotectant before immunohistochemical staining.

For doublecortin (DCX; a marker of newly born neurons) and Ki-67 (a cellular marker for proliferation) immunostaining, sections were first incubated with 3% H_2_O_2_ for 10 min to block endogenous peroxidase activity. After washing in PBS containing 0.2% Triton-X100 (PBS-T) (3 ×5 min), the sections were treated for 30 min with blocking solution (3% normal horse serum, 2% bovine serum albumin in PBS with Tween 20 (PBS-T; Sigma-Aldrich)). Sections were then incubated overnight at room temperature with the primary antibody (1:5000 in immunobuffer; DCX: guinea-pig anti-DCX, AB2253, Millipore; Ki-67: rabbit anti-Ki-67, Ab1558, Abcam). After washing in PBS-T, sections were incubated for 2 h with biotinylated secondary antibody (1:1000; DCX: goat anti-guinea-pig, Ab7138, Abcam; Ki-67: goat anti-rabbit, W0117, Abcam). Sections were then washed again in PBS-T and incubated for a further 2 h in ExtrAvidin peroxidise (1:1000, Sigma-Aldrich) in PBS. After washing in PBS, colour development was achieved by incubating with 3,3′ diaminobenzidine tetrahydrochloride (Vector DAB kit SK-4100). Sections were then mounted onto slides and left overnight. A nuclear red counterstain was used to facilitate DCX and Ki-67 staining quantification. The sections were then dehydrated through graded alcohols, cleared in xylene, and coverslipped in DPX mountant (Sigma-Aldrich). Example images of the staining are given in Figures S3 and S4.

### 3.4 Cell quantification

Cell counts were acquired manually under a light microscope (Leica, Leitz Wetzlar Germany) at 25x magnification by an experimenter blind to treatment. Cell counts were taken from three stereotaxic levels across the dentate gyrus and performed in triplicate for each brain. The mean cell count for both left and right hemispheres was calculated and used to produce a total cell count per animal.

## 4 Drugs

Corticosterone (CORT; 10 mg/kg, Sigma Aldrich, UK) was dissolved in a 5% DMSO, 95% sesame oil mix, and administered by subcutaneous injection. FG7142 (5.0 mg/kg, Sigma Aldrich, UK) was dissolved in a 10% DMSO, 20% cremophor, 80% saline mix, and administered by intraperitoneal injection using a low-stress, non-restrained technique (Stuart and Robinson, 2015). Drugs were administered in a dose volume of 1 ml/kg.

## 5 Statistical analysis

Experiments 1 & 2: For JBT experiments, all behavioural measures were analysed by week, calculated by taking the average of the two probe test sessions for that week. The cognitive bias index (CBI) was used as a measure of judgement bias in response to the midpoint tone (Papciak and Rygula, 2017). CBI was calculated by subtracting the proportion of responses made on the low reward lever from the proportion of responses made on the high reward lever. This created a score between − 1 and 1, where negative values represent a negative bias and positive values a positive bias. Change in CBI from baseline was calculated (pre-drug week minus either drug- or withdrawal-week score) to take into account individual differences in baseline bias, and to identify directional changes caused by drug treatments. Response latencies and percentages of positive responses, omissions and premature responses were also analysed (see Table S2 for details of these). Mixed analysis of variance (ANOVAs) were performed with one repeated measure (WEEK) as the within-subjects factor and GROUP as the between-subjects factor. Paired t-tests or independent samples t-tests were performed as appropriate as post-hoc tests if significant effects were established.

Experiments 3 & 4: For the ABT data, choice bias was calculated based on the number of choices made for the treatment-paired substrate vs the total number of trials (treatment-paired substrate + control-paired substrate). A value of 50 was then subtracted from the choice bias score to give a %Choice bias where a bias towards the treatment-paired substrate gave a positive value and a bias towards the control-paired substrate gave a negative score. Latency and trials to criterion were recorded during pairing sessions to determine whether there were any nonspecific effects of treatment (e.g, sedation, anorexia). Analysis for each treatment used a one-sample t-test against a theoretical mean of 0% choice bias where 0% is equivalent to 15 choices for the treatment-paired substrate and 15 choices for the vehicle-paired substrate. Between-treatment comparisons were made using unpaired t-tests. Analyses of the choice latency and trials to criterion were made using mixed ANOVAs with TREATMENT as the within-subject and GROUP as the between-subject factor. Sucrose preference data was analysed using unpaired t-test. For cell quantification, all analyses are conducted using a repeated measures ANOVA (rmANOVA) and post-hoc Least Significant Difference tests when appropriate. Total counts in control vs. CORT-treated animals were analysed using unpaired t-tests.

For all experiments, Huynh-Feldt corrections were used to adjust for violations of the sphericity assumption, Levene’s test was used to correct for inequality of variances. All statistical tests were conducted using SPSS 24.0.0.0 for Windows (IBM SPSS Statistics) with α = 0.05. Results are reported with the ANOVA F-value (degrees of freedom, error) and p-value as well as any post-hoc p-values. All graphs were made using Graphpad Prism 7.04 for Windows (Graphpad Software, USA).

## 6 Results

### 6.1 Experiment 1

**Fig. 2:**
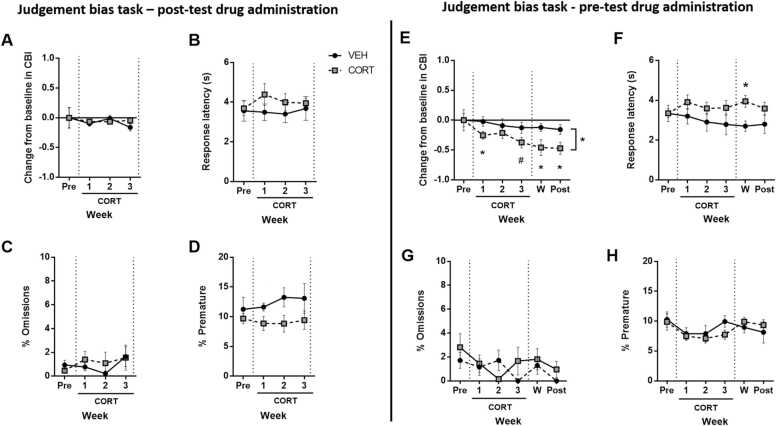
The effect of chronic treatment with corticosterone on the judgement bias task. Rats assigned to the chronic corticosterone (CORT) group received subcutaneous injections of CORT (10 mg/kg) daily for three weeks, whilst control rats received daily subcutaneous injections of 5% DMSO/95% sesame oil vehicle (VEH). For Experiment 1 (plots A-D), rats were injected at least three hours following the end of behavioural testing, whilst for Experiment 2 (plots E-H) rats were injected 30 min prior to behavioural testing. In both experiments, twice weekly test sessions (averaged) were conducted one week prior to treatment (x-axis label Pre) and for the three weeks during treatment (x-axis label CORT 1–3). The beginning and end of this three-week CORT treatment period is denoted by vertical dashed lines. For Experiment 2 only (plots E-H), twice weekly test sessions (averaged) were also conducted during the reduced-dosing withdrawal period (x-axis label W) and when CORT was no longer being administered (x axis label Post). There were no signiﬁcant differences between groups in either experiment during the pre-drug treatment period. (A) Chronic CORT treatment after testing had no effect on cognitive bias index (CBI). (B-D) There was also no significant effect of CORT on response latency (B) or percentage omissions (C) for the midpoint tone, or premature responses (D). (E) Chronic CORT treatment prior to testing caused cognitive bias index (CBI) to become more negative across the treatment period, and this was maintained during CORT withdrawal. (F) Response latency for the midpoint tone became slower in the CORT-treated group in the first week of withdrawal. (G) There was no effect on percentage omissions, or (H) premature responding. Data shown are for the midpoint tone only, and represent mean ± SEM. VEH group: n = 8, CORT group: n = 8. *p < 0.05; #p ≤ 0.06.

Administering daily CORT after behavioural testing did not cause a change in CBI (Fig. 2A). There was also no effect on response latencies or omissions for the midpoint tone (Fig. 2B and C). In the CORT-treated group, there was an apparent reduction in premature responses in weeks 1 and 3 but this failed to reach significance at p < 0.05 (GROUP: F_1,14_ = 3.765, p = 0.073; post hoc: ps ≤ 0.060; Fig. 2D, n = 8/group). There was no effect on behavioural measures for the high or low reward tones (Fig. S3).

## 7 Experiment 2

Administration of CORT prior to testing in the JBT induced a negative shift in CBI scores in the CORT-treated group (n = 8) compared to the control group (n = 8). This was initially apparent in the first week of treatment, with the effect increasing over the drug treatment period, and maintained throughout the drug withdrawal period (WEEK: F_2.726,38.165_ = 3.135, p = 0.041; and GROUP: F_1,14_ = 6.249, p = 0.025, post-hoc comparisons between groups: drug week 1: p = 0.039, drug week 3: p = 0.071, withdrawal week 1: p = 0.039, withdrawal week 2: p = 0.028; Fig. 2E). The CORT group also exhibited a more negative change in CBI in drug and withdrawal weeks compared to the pre-drug week (one sample t-tests for CORT group only: ps ≤ 0.051; Fig. 2E). This treatment protocol also caused an increase in response latencies for the midpoint tone only (WEEKxGROUP: F_5,70_ = 2.531, p = 0.037, and post-hoc withdrawal week 1: p = 0.007; Fig. 2F). Irrespective of treatment, main effects of WEEK revealed that, for both high and low tones, animals responded faster across the duration of the experiment (high tone: F_3.113,43.582_ = 7.600, p < 0.001; low tone: F_4.297,60.158_ = 2.700, p = 0.035; Fig. S4A). Administration of CORT prior to testing did not alter omissions (Fig. 2G & Fig. S4C) or premature responding (Fig. 2H).

## 8 Experiment 3

**Fig. 3:**
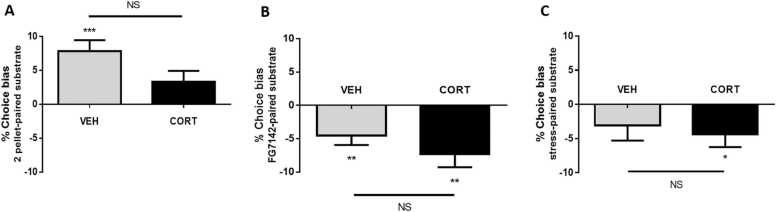
The effects of chronic corticosterone (CORT) treatment on affective biases in Experiment 3. In the ABT, CORT treatment was found to: A) attenuate reward-induced positive affective bias, but had no effect on negative affective biases induced by B) FG7142, or C) psychosocial stress. Data is presented as mean ± SEM (VEH: n = 14 and CORT: n = 16). *p < 0.05, * *p < 0.01, * **p < 0.001 vs. 0% choice bias, NS: p > 0.05.

The results from the reward-induced positive bias study show that, while vehicle-treated animals (n = 14) demonstrated a significant positive bias towards the cue associated with the higher value reward, animals treated with daily injections of 10 mg/kg CORT (n = 16) failed to do so (Fig. 3A). The difference between the two treatment groups however failed to reach statistical significance (unpaired t-test: t_28_ = 1.97, p = 0.059). When treated with FG7142, both treatment groups developed a significant negative affective bias (Fig. 3B), and although the magnitude of negative bias appeared to be larger in CORT-treated animals, this did not reach statistical significance (unpaired t-test: t_28_ = 1.1, p = 0.28). FG7142 also induced a significant increase in choice latency in both groups of animals compared to vehicle treatment (TREATMENT: F_1,28_ = 14.5, p = 0.0007; Table S3). In the final manipulation, when subjected to restraint stress and social isolation, only CORT-treated animals demonstrated a significant negative affective bias (Fig. 3C) in the ABT. Again, post-hoc testing showed there was no significant group difference (unpaired t-test: t_28_ = 0.45, p = 0.66).

## 9 Experiment 4

### 9.1 Behavioural testing

**Fig. 4:**
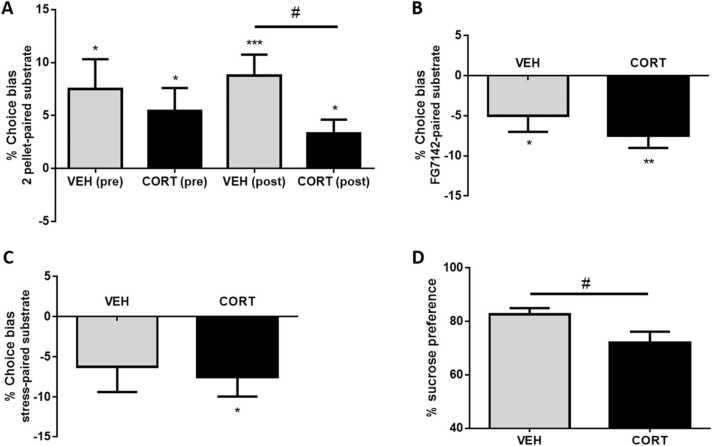
The effect of chronic corticosterone treatment and social isolation (CORT+SI) on affective biases, and sucrose preference in Experiment 4. The CORT+SI procedure was found to: A) attenuate reward-induced positive affective bias in the ABT, but had no significant effect on negative affective biases induced by B) FG7142, or C) psychosocial stress. However D) sucrose preference was reduced in the treatment group compared to controls. Data is presented as mean ± SEM (n = 8/group). *p < 0.05, ***p < 0.001 vs. 0% choice bias, #p < 0.05 vs. VEH.

Before the chronic CORT procedure, both treatment groups exhibited a significant reward-induced bias towards the higher value cue (Fig. 4 A) and there was no significant difference between the groups (unpaired t test: t_14_ = 0.59, p = 0.57). Following 6 weeks of CORT+SI treatment, animals still demonstrated this reward-induced bias, however it was significantly reduced compared to the vehicle-treated group (unpaired t test: t_14_ = 2.3, p = 0.037, n = 8/group). Analysis of the pairing session data reveals that vehicle-treated animals had faster choice latencies during high value reward pairing sessions compared to the low reward sessions, whereas CORT+SI animals showed no difference (TREATMENT: F1_,14_ = 1.75, p = 0.21; Table S4). Treatment with FG7142 induced a negative affective bias in both vehicle and CORT+SI groups (Fig. 4B), but there was no significant difference in the magnitude of the bias between groups (unpaired t-test: t_14_ = 1.0, p = 0.33). Analysis of the choice latency data shows that CORT+SI animals had significantly longer choice latencies during treatment-pairing sessions compared to vehicle animals (F_1,14_ = 5.2, p = 0.0387; Table S4). These animals also required significantly more trials to reach criterion in pairing sessions following FG7142 treatment compared to control pairing sessions and compared to the VEH group (TREATMENTxGROUP: F_1,14_ = 4.6, p = 0.0496; Table S4). While the 30 min period of restraint stress failed to induce a significant affective bias in vehicle-treated animals, CORT+SI animals showed a significant negative affective bias (Fig. 4 C), however there was no significant group difference (unpaired t-test: t_14_ = 0.32, p = 0.76). In the SPT (Fig. 4D), CORT+SI induced a significant reduction in sucrose preference (unpaired t-test: t_14_ = 2.2, p = 0.043).

### 9.2 Immunostaining for neurogenesis

**Fig. 5:**
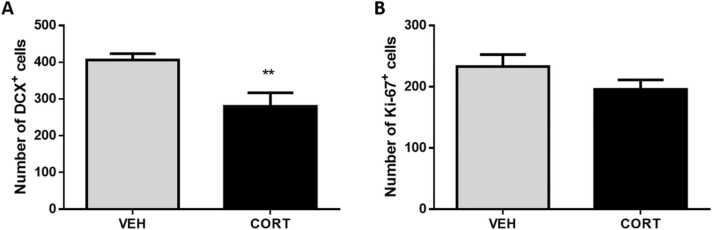
The effect of chronic CORT and social isolation (CORT+SI) on the number of (A) DCX+ and (B) Ki-67 + cells in the dentate gyrus. The CORT+SI procedure induced a decreased in DCX+ cells in the dentate gyrus, but had no effect on the number of Ki-67 + cells. Data is presented as mean ± SEM (n = 8/group). * *p < 0.01 vs. VEH.

There was a significant main effect of treatment on DCX expression in the dentate gyrus (F_1,14_ = 9.68, p = 0.008), and post-hoc analysis revealed that the chronic CORT+SI procedure induced a decreased in DCX+ cells in this region (unpaired t test: t_14_ = 3.112, p = 0.008 vs VEH; Fig. 5A). Overall the total number of Ki67 + cells in the dentate gyrus was unaffected (t14 = 1.00, p = 0.336 vs VEH (Fig. 5B). Representative images from the DCX and Ki67 immunohistochemistry are given in Figures S3 and S4.

## 10 Discussion

In the present study we show that chronic treatment with the stress hormone CORT induces specific behavioural and neurobiological changes that are consistent with a prolonged period of negative affect. The CORT treatment induced a negative decision-making bias in the JBT but only when animals experienced elevated levels of CORT during the behavioural procedure. Consistent with our findings in the early life adversity model (Stuart et al., 2019), we observed impaired reward learning and memory in the RLA. There was some evidence that sensitivity to acute negative affective state manipulations was increased in the chronic CORT-treated animals, but clear group differences were not observed. Using an immunohistochemical marker of neurogenesis, we found that the treatment protocol decreased the expression of DCX in the dentate gyrus, indicating reduced hippocampal neurogenesis. The following discussion considers how these neuropsychological deficits resulting from chronic CORT compare with findings in our early life adversity model and the wider implications for studying depression-related neurobiology in rodents.

Similar to previous findings using chronic psychosocial stressors (Hales et al., 2016, Papciak et al., 2013), chronic CORT treatment induced a negative bias in interpretation of ambiguous information, but only when CORT was administered prior to JBT testing. The immediate negative effect on bias of CORT treatment in the first week mirrors the acute negative effect of combined CORT and reboxetine treatment on bias reported by Enkel et al. (2010), and an acute CORT treatment alone (Hales et al., 2021). This negative bias was maintained throughout the chronic treatment period. It appears that the negative bias became more pronounced by the end of the treatment period, and was sustained post withdrawal. This suggests that the effects are not dependent on current exposure to CORT, but that chronic CORT treatment induces a long-lasting negative affective bias.

The same chronic CORT treatment had no effect when given after testing sessions, and CORT treatment given prior to JBT testing did not alter responding to the reference cues. The reward magnitude associated with reference cues was learnt prior to chronic CORT treatment, and did not alter during treatment, whereas reward outcome for the ambiguous cue is not learnt, and choice is hypothesised to be driven by online decision-making processes that can be influenced by affective state (Mendl et al., 2009). The contrast between effects dependent on the timing of administration of CORT suggests that the negative bias observed requires the acute neuropsychological effects of the drug to be present during the task experience. As these effects are then sustained post-treatment, this suggests a possible role for learning with long term effects on memory consistent with a neuropsychological hypothesis of depression (Godlewska and Harmer, 2021, Harmer et al., 2009, Roiser et al., 2012). The dissociation of the effects based on timing suggests that these decision-making biases involve both biological and psychological factors, requiring experience-dependent learning under the influence of the affective state. These effects were sustained after treatment ended suggesting this learning can then have ongoing effects on decision-making behaviour.

In the ABT experiments, both the CORT treatment alone, and combined with social isolation failed to cause any robust effects on the vulnerability of the animals to negative affective biases induced by either the anxiogenic compound FG7142, or restraint stress administered prior to learning. This finding contrasts our previous observations using an early life adversity model, where animals showed an exaggerated negative affective bias in response to acute stress (Stuart et al., 2019). This may suggest that there are fundamental differences between the chronic CORT and early life adversity models in terms of their effects on altering sensitivity to acute negative biases, or perhaps reflect a reduced ability for FG7142 to induce a robust negative bias compared to acute CORT as we have used in the early life adversity model. There may also be a potential greater variability in this model of depression and hence a larger n number might be needed for this type of interaction study.

Using the RLA, we are able to investigate the effects of CORT treatment on reward‐related learning and memory and their effects on subsequent reactivation of the reward-associated cues. In this task normal animals will show a positive bias towards the digging substrate that they have previously learned to associate with a higher value of reward (Stuart et al., 2013) and we propose that a loss or reduction of this bias reflects a failure in reward learning. In the present study we show that both CORT treatment alone, or the combined CORT&SI procedure impaired this reward-induced positive bias. This deficit appears to be a common finding in animals that have been subjected to chronic treatment with putative pro-depressant drugs (i.e. drugs that are known to increase risk of negative affective states in humans) (Lydall et al., 2010, Sahin et al., 2016, Stuart et al., 2017), as well as animals that have undergone early life adversity (Stuart et al., 2019). There is also evidence from both human and animal studies which suggest impairments in probabilistic reward learning tasks which may involve similar underlying neurobiology to the findings presented here (Der-Avakian et al., 2016).

The reduction is sucrose preference we observed following chronic treatment with CORT is a finding consistent with previous literature demonstrating that repeated CORT injections result in reduced sucrose consumption (Bai et al., 2018, Gregus et al., 2005, Li et al., 2015). Deficits in sucrose preference have been interpreted as representing anhedonia, one of the key symptoms of depression (DSM-V, American Psychiatric Association, 2013). However not all rodent models of depression have been found to produce this deficit (Der-Avakian et al., 2016, Stuart et al., 2019), including our own work using the early life adversity model in rats where neuropsychological impairments as measured in the ABT and JBT occurred in the absence of a change in sucrose preference (Stuart et al., 2019). We propose that the SPT measures only one aspect of reward processing: consummatory behaviour, and that this behaviour may be differentially affected by different affective manipulations, with the reward learning deficits representing a more consistent deficit across depression models (Slaney et al., 2018, Lewis et al., 2019).

Overall, this study demonstrates that chronic stress, a key risk factor for MDD, induces negative decision-making biases and impaired reward learning in the JBT and ABT respectively, providing further evidence for the potential significance of neuropsychological deficits in development of MDD. These findings also concur with findings from both patients and other rodent model of depression and suggest negative affective biases provide a translational, cognitive biomarker and these biases may play an important role in the development and perpetuation of MDD. The studies were limited to male animals and further studies in female animals are needed to understand if there are sex-related differences in the neuropsychological effects seen in this model.

## Funding

This research was funded by an Industrial Partnership Award awarded by Biotechnology and Biological Sciences Research Council in collaboration with Boehringer Ingelheim, UK' (BBSRC) (Grant no: BB/N015762/1) and carried out with intellectual support from Boehringer Ingelheim, and a 10.13039/501100000265Medical Research Council, UK' (MRC) project grant (Grant no: MR/L011212/1).

## CRediT authorship contribution statement

Claire A. Hales and Sarah A. Stuart performed the research, analysed data, co-wrote and edited the paper. Jennifer Griffiths and Julia Bartlett performed research and analysed data. Roberto Arban and Bastian Hengerer designed the research and edited the paper. Emma SJ Robinson designed the research and wrote and edited the paper.

## Declaration of interest

ESJR has current or has previously obtained research grant funding through PhD studentships, collaborative grants, and contract research from Boehringer Ingelheim, COMPASS Pathways, Eli Lilly, MSD, Pfizer and SmallPharma. The authors declare no conflict of interest.
