# Supplementary material for: Investigating neuropsychological and reward-related deficits in a chronic corticosterone-induced model of depression
Source: Psychoneuroendocrinology. 2023 Jan;147:105953. doi: 10.1016/j.psyneuen.2022.105953 (PMC10465973; doi:10.1016/j.psyneuen.2022.105953)
Supplement: Supplementary file 1 — Supplementary material [file mmc1.docx]

**SUPPLEMENTARY TABLES**

***Table S1 – Training stages and required performance criteria for the judgement bias task.***

| **Stage**  **1 – Magazine training** | **Description** |  | **Criteria** | **Sessions to meet criteria** | |
| --- | --- | --- | --- | --- | --- |
|  | Tone (2 kHz only for half the session followed by 8 kHz only for the rest of the session, order counterbalanced across rats) played for 20 s followed by release of one pellet into magazine; 10 s ITI. No levers available. |  | 20 pellets eaten for each tone frequency | Cohort 1  1 | Cohort 2  1 |
| **2 – Tone training** | Response on lever during tone (2 kHz or 8kHz only, order counterbalanced across rats) rewarded with one pellet. Lever corresponding to that tone frequency available only. |  | > 50 trials completed for two consecutive sessions on each tone frequency | 4 | 4 |
| **3 – Discrimination training** | Response on correct corresponding lever only during tone (either 2 kHz or 8 kHz presented pseudorandomly) rewarded with one pellet. Both levers available. |  | > 70% accuracy for both tones, no significant differences on analysed behavioural measures over three sessions and < 1:1 ratio of correct:premature responses | 15 | 10 |
| **4 – Reward magnitude training** | As Stage 3 but response on correct corresponding lever only rewarded with four pellets for high reward tone and one pellet for low reward tone. Both levers available. |  | As for Stage 3 but with > 60% accuracy for both tones  (to allow for biases in responding to reference tones caused by the difference in associated reward magnitude). | 9 | 9 |

*Training sessions consisted of 100 trials. Apart from where otherwise specified in the description, for all sessions (training, baseline and probe test sessions), response levers were extended at the beginning of every session and remained extended for the duration of the session (maximum one hour). During sessions (where applicable) pressing the incorrect lever during a tone was punished by a 5 s timeout, as was an omission if the rat failed to press any lever during the 20 s tone. Tone presentations were separated by an inter-trial interval of 5 s, during which time premature responses on either lever were punished by a 5 s timeout. During a timeout, the house light was illuminated, and responses made on levers were recorded but had no programmed consequences.*

***Table S2 - Description of statistical analysis for other behavioural measures***

| **Behavioural Measure**  **Response latency** | **Analysed for:** |  | **Description** | **Statistical analysis** |
| --- | --- | --- | --- | --- |
|  | Each tone |  | Time between presentation of the tone and response on the lever (correct lever for high and low reward tones, either lever for midpoint tone) | Two-way repeated measures ANOVA with tone and session as within-subjects factors |
| **Accuracy** | Reference tones (high and low tones) |  | > 50 trials completed for two consecutive sessions on each tone frequency |  |
| **Percentage omissions** | Each tone |  | Number of trials where no lever press occurred during 20 s tone presentation divided by total completed trials for that tone |  |
| **Percentage of premature responses** | Whole session |  | Number of trials where a response was made in the 5 s inter-trial interval divided by total completed trials | Repeated measures ANOVA with session as the within-subjects factor |

*This table details the other behavioural measures (apart from cognitive bias index) that were analysed for each experimental manipulation and are presented in the supplementary figures. ANOVA – analysis of variance.*

***Table S3 – ABT pairing session data showing choice latency and trials to criterion in rats chronically treated with corticosterone (CORT) or vehicle (VEH).***

| **Study** | **Group** | **Choice Latency (sec)** | |  | **Trials to criterion** | |
| --- | --- | --- | --- | --- | --- | --- |
|  |  | **1 pellet** | **2 pellets** |  | **1 pellet** | **2 pellets** |
| **2 vs 1** | VEH | 2.0±0.2 | 1.9±0.1 |  | 6.6±0.3 | 6.6±0.3 |
|  | CORT | 1.8±0.1 | 1.9±0.1 |  | 7.3±0.4 | 6.6±0.3 |
|  |  |  | |  |  | |
| **Study** | **Group** | **Choice Latency (sec)** | |  | **Trials to criterion** | |
|  |  | **Control** | **Treatment** |  | **Control** | **Treatment** |
| **FG 7142** | VEH | 3.0±0.3 | **4.6±0.7*** |  | 8.1±0.4 | 8.9±0.4 |
|  | CORT | 2.6±0.4 | **3.9±0.5**** |  | 8.0±0.4 | 7.7±0.6 |
| **Restraint stress &** | VEH | 2.5±0.2 | 3.0±0.4 |  | 8.1±0.6 | 6.9±0.4 |
| **social isolation** | CORT | 2.9±0.3 | 3.1±0.3 |  | 8.1±0.2 | 7.8±0.4 |

*Data is presented as mean ± SEM choice latency and number of trials to criterion during treatment- vs. control-paired sessions. *p < 0.05, **p < 0.01 vs. control sessions (n=8/group)*

***Table S4 – ABT pairing session data showing choice latency and trials to criterion in rats chronically treated with corticosterone and social isolation (CORT+SI) or vehicle (VEH).***

| **Study** | **Group** | **Choice Latency (sec)** | |  | **Trials to criterion** | |
| --- | --- | --- | --- | --- | --- | --- |
|  |  | **1 pellet** | **2 pellets** |  | **1 pellet** | **2 pellets** |
| **2 vs 1** | VEH | 5.6±0.8 | 6.1±0.8 |  | 9.9±0.9 | 8.8±0.7 |
| **pre-CORT+SI** | CORT+SI | 5.8±0.4 | 6.0±0.5 |  | 7.6±0.4 | 9.8±1.0 |
| **2 vs 1** | VEH | 4.0±0.3 | **3.3±0.2*** |  | 7.8±0.5 | 8.2±0.4 |
| **post-CORT+SI** | CORT+SI | 4.3±0.4 | 4.0±0.4 |  | 7.6±0.3 | 7.4±0.3 |

| **Study** | **Group** | **Choice Latency (sec)** | |  | **Trials to criterion** | |
| --- | --- | --- | --- | --- | --- | --- |
|  |  | **Control** | **Treatment** |  | **Control** | **Treatment** |
| **FG 7142** | VEH | 6.0±0.5 | 5.4±0.3 |  | 8.0±0.4 | 7.8±0.5 |
|  | COR+TSI | 7.6±0.9 | **7.6±0.7#** |  | 7.9±0.4 | **9.5±0.4#*** |
| **Restraint** | VEH | 4.7±0.5 | 5.1±0.6 |  | 7.4±0.5 | 6.9±0.3 |
| **stress** | CORT+SI | 4.3±0.5 | 4.8±0.4 |  | 7.0±0.3 | 7.4±0.4 |

*Data is presented as mean ± SEM choice latency and number of trials to criterion during treatment- vs. control-paired sessions. *p < 0.05 vs. control sessions, #p < 0.05 vs VEH group (n=8/group).*

**SUPPLEMENTARY FIGURES**

**Supplementary Figure 1: The effect of chronic treatment with corticosterone administered before testing on judgement bias.**

**
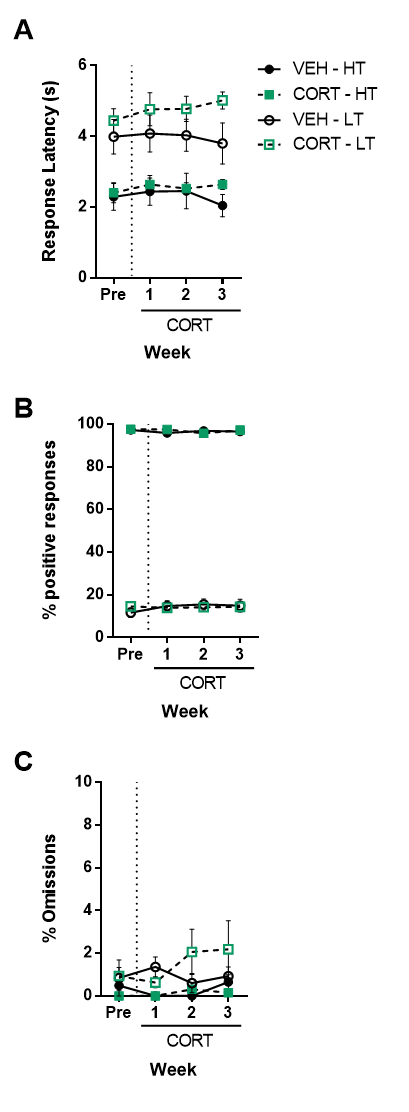
**Rats assigned to the chronic corticosterone (CORT) group experienced subcutaneous injections of CORT (10 mg/kg) daily for three weeks, whilst control rats experienced daily subcutaneous injections of 5% DMSO/95% sesame oil vehicle (0.0 mg/kg). Rats were injected at least three hours following the end of behavioural testing. Twice weekly test sessions (averaged) were conducted one week prior to treatment (Pre) and for the three weeks during treatment (CORT 1–3). There were no signiﬁcant differences between groups during the pre-drug treatment period. (A) There was no effect of chronic CORT treatment on response latencies for the high or low reward tones. (B) There was no effect on percentage positive responding. (C) Omissions did not change during chronic CORT treatment. Data represent mean ± SEM. Control group: n=8, CORT group: n=8. HT - high reward tone; LT - low reward tone.

**Supplementary Figure 2: The effect of chronic treatment with corticosterone administered before testing on judgement bias.**

**
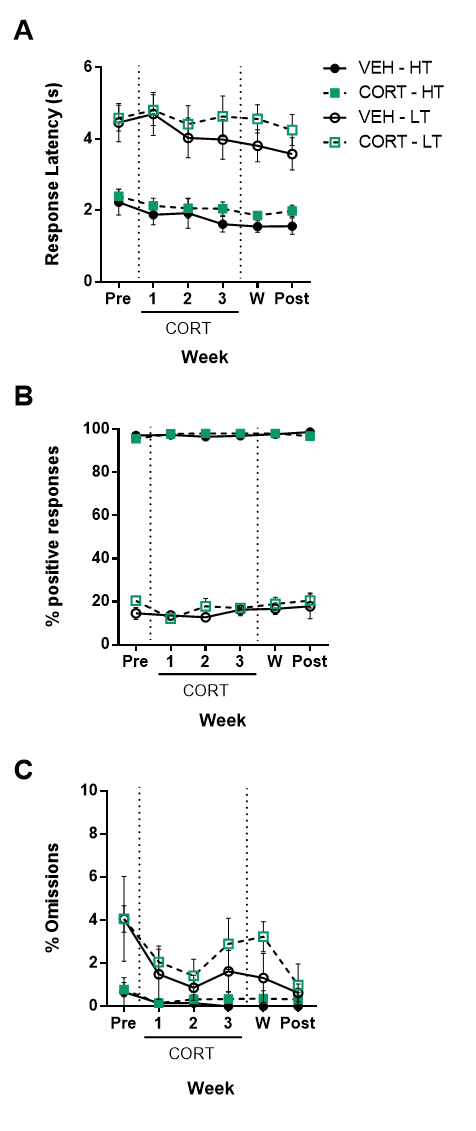
**Rats assigned to the chronic corticosterone (CORT) group experienced subcutaneous injections of CORT (10 mg/kg) daily for three weeks, whilst control rats experienced daily subcutaneous injections of 5% DMSO/95% sesame oil vehicle (0.0 mg/kg). Rats were injected 30 minutes prior to behavioural testing. Twice weekly test sessions (averaged) were conducted one week prior to treatment (Pre) and for the three weeks during treatment (CORT 1–3). There were no signiﬁcant differences between groups during the pre-drug treatment period. (A) Irrespective of dose, there was a main effect of session for both high reward (*F_3.113,43.582_* = 7.600, *p* < 0.001) and low reward (*F_4.297,60.158_* = 2.700, *p* = 0.035) tones, indicating that all rats became quicker to response to these tones across time. (B) There was no effect of chronic CORT treatment on responding to the high or low reward tones. (C) There was a main effect of CORT for percentage omissions (*F_3.607,50.494_* = 5.359, *p* = 0.002), and a significant post-hoc comparison between the Pre week and 1^st^ CORT treatment week (*p* = 0.031), showing that for both groups omissions decreased. Data represent mean ± SEM. Control group: n=8, CORT group: n=8. *** *p* < 0.001, **p* < 0.05. HT - high reward tone; LT - low reward tone.

*
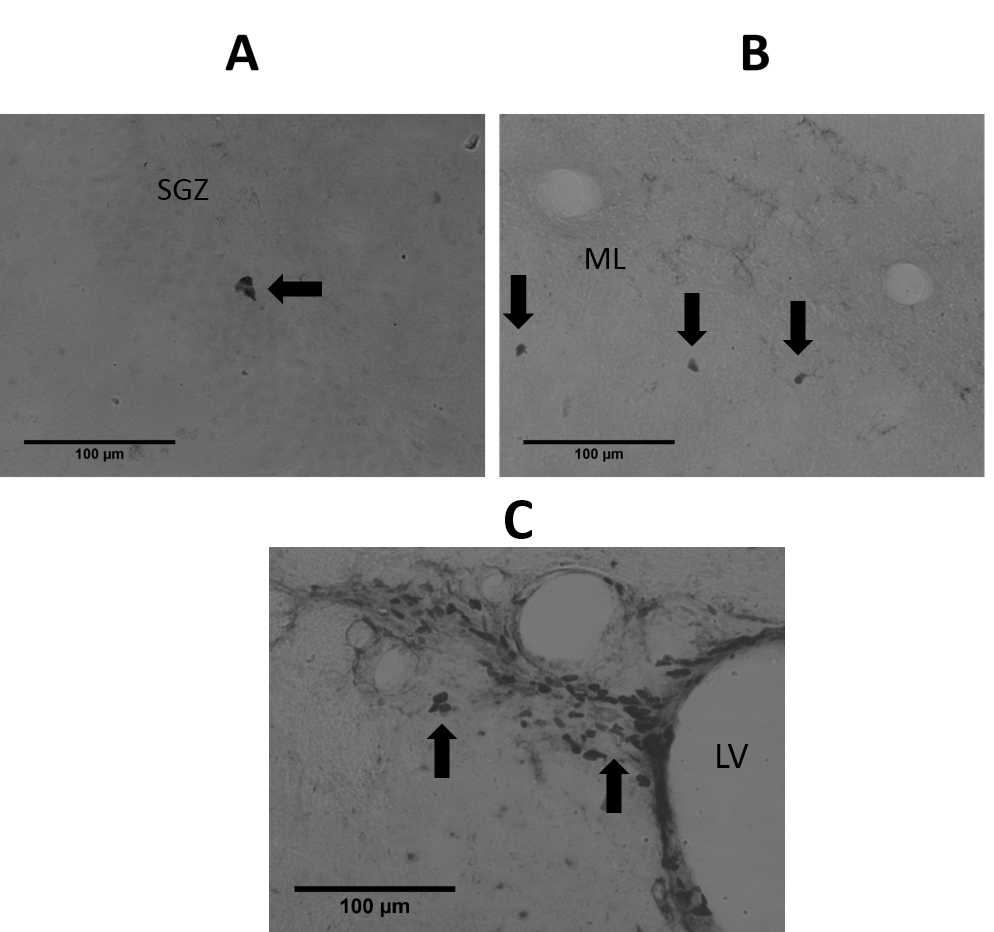
*

Supplementary figure 3: Images of Ki-67+ cells within the dentate gyrus of the hippocampus

Arrows indicate examples of Ki-67+ cells. Image A shows Ki-67+ cells within the SGZ of the DG. Image B shows Ki-67 cells within the ML of the DG and image C shows Ki-67+ cells that are extending from the SVZ of the lateral ventricle (LV) (scale bar =100 µm).

Abbreviations: GCL: granular cell layer, HIL: hilus, LV: lateral ventricle ML: molecular layer, SGZ: subgranular zone, SVZ: subventricular zone.


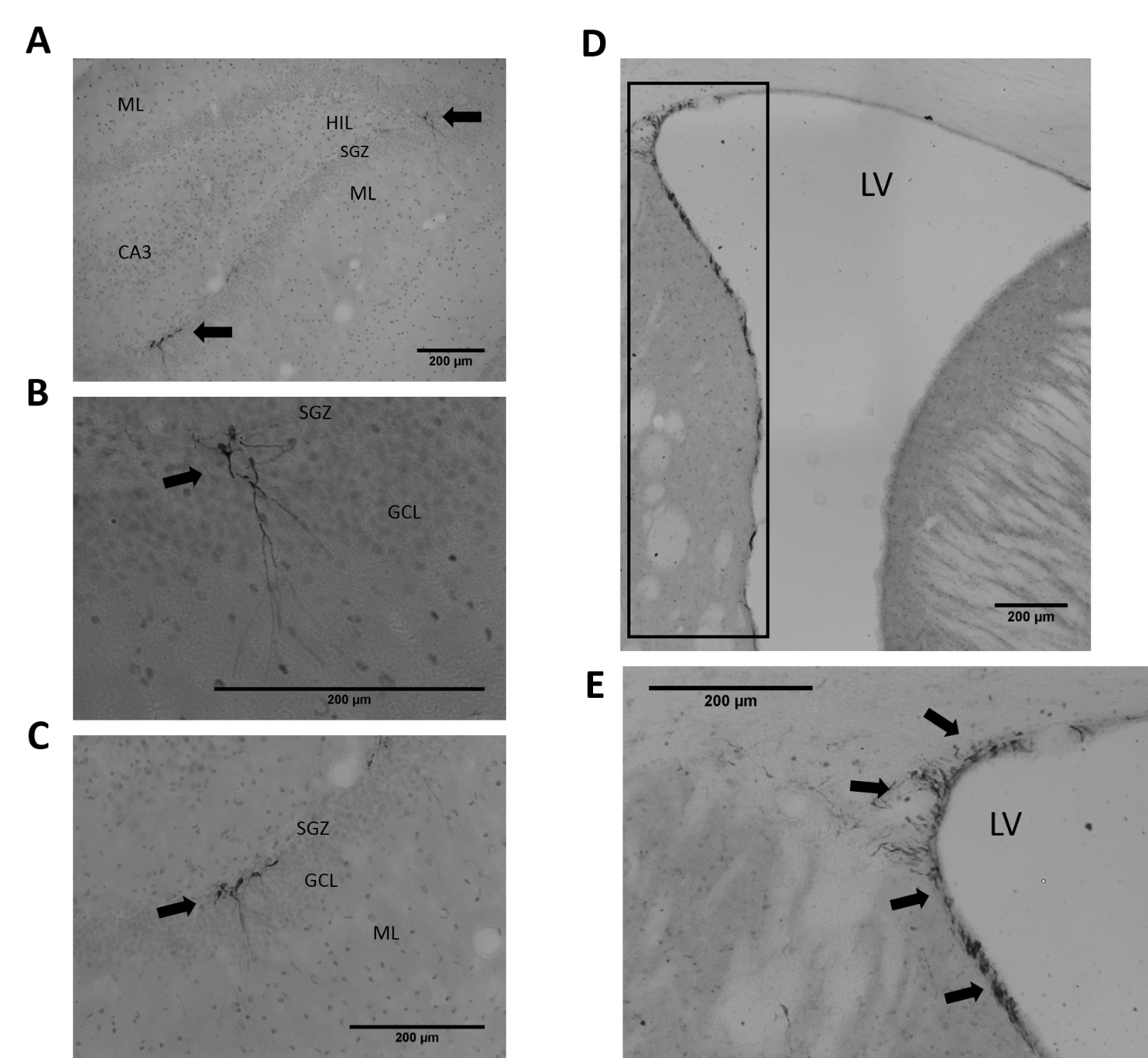


Supplementary figure 4: Images of DCX+ neurones within the dentate gyrus of the hippocampus.

Arrows indicate examples of DCX+ cells. Image A shows the dorsal DG. Within this image, there are 2 regions of DCX+ neurons which reside within the SGZ. Images B and C are enlargements of these 2 regions. Along the border of the LV there are DCX+ neurones within the SVZ (image D). Image E shows an enlargement of the upper left hand corner of the lateral ventricle (scale bar = 200µm)

Abbreviations: GCL: granular cell layer, HIL: hilus, LV: lateral ventricle ML: molecular layer, SGZ: subgranular zone, SVZ: subventricular zone.
